# Impaired Insulin Signaling Mediated by the Small GTPase Rac1 in Skeletal Muscle of the Leptin-Deficient Obese Mouse

**DOI:** 10.3390/ijms241411531

**Published:** 2023-07-16

**Authors:** Man Piu Chan, Nobuyuki Takenaka, Takaya Satoh

**Affiliations:** Laboratory of Cell Biology, Department of Biological Chemistry, Graduate School of Science, Osaka Metropolitan University, Sakai 599-8531, Japan; md304019@st.osakafu-u.ac.jp (M.P.C.); nobu.takenaka@omu.ac.jp (N.T.)

**Keywords:** Akt2, glucose uptake, GLUT4, GTPase, insulin, obesity, Rac1, RalA, skeletal muscle

## Abstract

Insulin-stimulated glucose uptake in skeletal muscle is mediated by the glucose transporter GLUT4. The small GTPase Rac1 acts as a switch of signal transduction that regulates GLUT4 translocation to the plasma membrane following insulin stimulation. However, it remains obscure whether signaling cascades upstream and downstream of Rac1 in skeletal muscle are impaired by obesity that causes insulin resistance and type 2 diabetes. In an attempt to clarify this point, we investigated Rac1 signaling in the leptin-deficient (*Lep^ob/ob^*) mouse model. Here, we show that insulin-stimulated GLUT4 translocation and Rac1 activation are almost completely abolished in *Lep^ob/ob^* mouse skeletal muscle. Phosphorylation of the protein kinase Akt2 and plasma membrane translocation of the guanine nucleotide exchange factor FLJ00068 following insulin stimulation were also diminished in *Lep^ob/ob^* mice. On the other hand, the activation of another small GTPase RalA, which acts downstream of Rac1, by the constitutively activated form of Akt2, FLJ00068, or Rac1, was partially abrogated in *Lep^ob/ob^* mice. Taken together, we conclude that insulin-stimulated glucose uptake is impaired by two mechanisms in *Lep^ob/ob^* mouse skeletal muscle: one is the complete inhibition of Akt2-mediated activation of Rac1, and the other is the partial inhibition of RalA activation downstream of Rac1.

## 1. Introduction

Insulin-stimulated glucose uptake in skeletal muscle is mediated by the translocation of the glucose transporter GLUT4 from GLUT4 storage vesicles to the plasma membrane [1,2,3,4,5]. Insulin induces the intracellular trafficking of GLUT4-containing vesicles, ultimately leading to the fusion of the vesicles with the plasma membrane. Following this, GLUT4 is redistributed to the cell surface and permits blood glucose to enter the cell across the plasma membrane. In response to insulin stimulation, diverse signaling pathways downstream of the insulin receptor that control the plasma membrane translocation of GLUT4 are activated [1,2,3,4,5]. In particular, a kinase cascade composed of phosphoinositide 3-kinase (PI3K) and downstream protein kinases, including PDK1 and Akt2, plays a pivotal role in the signaling mechanisms.

Recent studies have demonstrated that the Rho family small GTPase Rac1 also acts as a key component in the regulation of glucose uptake following insulin stimulation in skeletal muscle [4,5,6,7,8,9,10,11,12]. Originally, the involvement of Rac1 in this signaling was proposed by studies in cultured myoblasts and in vitro differentiated myotubes [7,8,9,11]. This notion was further supported by evidence obtained from in vivo experiments in mouse models [10,12]. In fact, muscle-specific *rac1* knockout mice showed impaired glucose tolerance and higher plasma insulin concentrations after intraperitoneal glucose injection [10]. Therefore, the role of Rac1 in the regulation of glucose uptake in skeletal muscle is physiologically important for the maintenance of glucose homeostasis.

The detailed mechanisms for Rac1-mediated regulation of glucose uptake have also been explored in myoblast cell lines and mouse skeletal muscle. The ectopic expression of a constitutively activated mutant of PI3K or Akt2 caused the activation of Rac1 in L6 myoblasts and mouse gastrocnemius muscle fibers [13,14,15]. Additionally, the plasma membrane translocation of GLUT4 was induced by the ectopic expression of these constitutively activated mutants in gastrocnemius muscle fibers of wild-type, but not muscle-specific *rac1* knockout, mice [14]. Finally, small interfering RNA-mediated knockdown of Akt2 significantly reduced the activation of Rac1 following insulin stimulation or the ectopic expression of a constitutively activated mutant of PI3K [16]. Therefore, we proposed that Rac1 acts downstream of Akt2, regulating GLUT4 translocation. In contrast, it has been reported that Rac1 serves as a downstream component of PI3K, but not Akt2, and Akt2 and Rac1 regulate intracellular trafficking of GLUT4-containing vesicles and cytoskeletal rearrangements, respectively [5,6,17,18].

The Dbl family guanine nucleotide exchange factor (GEF) FLJ00068 (also termed PLEKHG4 or puratrophin-1) has been implicated as a direct regulator of Rac1 downstream of Akt2 in skeletal muscle insulin signaling [11,14,19,20]. In response to insulin stimulation, FLJ00068 was translocated to the plasma membrane in mouse gastrocnemius muscle fibers [20]. This may explain, at least in part, the mechanisms underlying the Akt2-dependent activation of FLJ00068.

The small GTPase RalA is a member of the Ras family, and its role in the regulation of GLUT4 translocation was reported originally in adipocytes [21]. Likewise, RalA regulates insulin-stimulated GLUT4 translocation in skeletal muscle [16,22,23]. Remarkably, RalA functions downstream of Rac1 in skeletal muscle, although the detailed mechanisms for Rac1-dependent activation of RalA remain to be solved [16,22,23].

As described above, accumulating evidence suggests the important role of Rac1 in insulin-stimulated glucose uptake in skeletal muscle. Although it is well known that obesity causes insulin resistance of skeletal muscle, it remains unclear whether Rac1-mediated signaling is indeed impaired in insulin-resistant states associated with obesity. Moreover, it is important to clarify the mechanisms whereby signal transduction cascades upstream and downstream of Rac1 are affected by obesity. Therefore, we herein employed the leptin-deficient obese (*Lep^ob/ob^*) mouse model [24] and assessed the effects of obesity on Rac1-mediated insulin signaling in skeletal muscle.

## 2. Results

### 2.1. Inhibition of Insulin-Stimulated Rac1 Activation in Lep^ob/ob^ Mouse Skeletal Muscle

Accumulating evidence suggests that Rac1 is a key regulator of insulin-stimulated GLUT4 translocation to the plasma membrane in skeletal muscle [4,5,6,7,8,9,10,11,12]. Therefore, it is important to clarify whether the Rac1 signaling pathway is negatively affected by obesity, which causes a variety of diseases, including type 2 diabetes. To this end, we investigated Rac1 signaling in detail in the skeletal muscle of the *Lep^ob/ob^* mouse model [24]. Twenty-four-week-old *Lep^ob/ob^* and control mice fed a normal chow diet were used in this study. Body weights of these *Lep^ob/ob^* and control mice were 63.2 ± 7.6 and 26.7 ± 3.6 (means ± S.E., *n* = 10) g, respectively.

**Figure 1:**
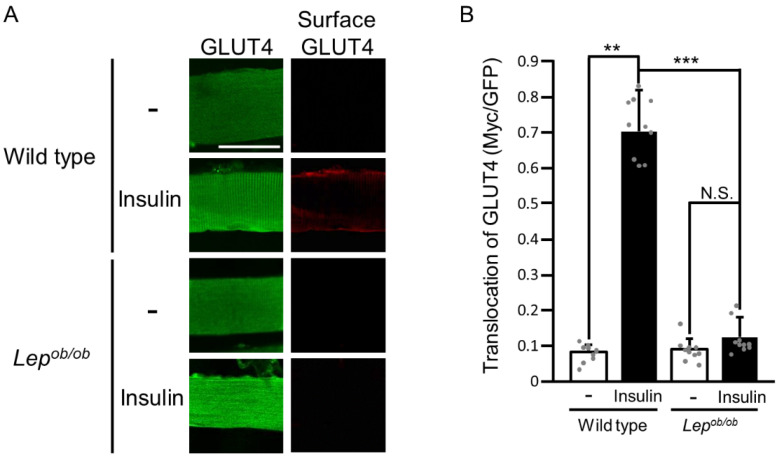
The translocation of GLUT4 to the plasma membrane following insulin stimulation in the gastrocnemius muscle of *Lep^ob/ob^* mice. (**A**) Insulin was administered by intravenous injection, and GLUT4 translocation in gastrocnemius muscle was assessed by using the exofacial epitope-tagged GLUT4 reporter GLUT4*myc*7-GFP. Total (green color) and cell surface-localized (red color) GLUT4*myc*7-GFP molecules in gastrocnemius muscle fibers were visualized. Scale bar, 50 μm. (**B**) Quantification of the translocation of GLUT4 to the plasma membrane (Myc/GFP, arbitrary units). Data are shown as means ± S.E. (*n* = 10). ** *p* < 0.01, *** *p* < 0.001, (Student’s *t*-test).

As a first step, we confirmed the insulin-resistant state of *Lep^ob/ob^* mouse gastrocnemius muscle by assessing insulin-stimulated GLUT4 translocation to the plasma membrane (Figure 1). We applied a GLUT4 reporter assay [25] to mouse gastrocnemius muscle fibers as previously described [12,14,16,19,20,23]. Only the GLUT4 reporter that was translocated to the plasma membrane was recognized by an anti-Myc tag antibody and was visualized by immunofluorescent microscopy (red color). The expression level of the GLUT4 reporter was estimated by the intensity of green fluorescence of the tagged green fluorescence protein (GFP). As expected, insulin-stimulated translocation of GLUT4 to the plasma membrane was markedly impaired in *Lep^ob/ob^* mouse gastrocnemius muscle (Figure 1).

**Figure 2:**
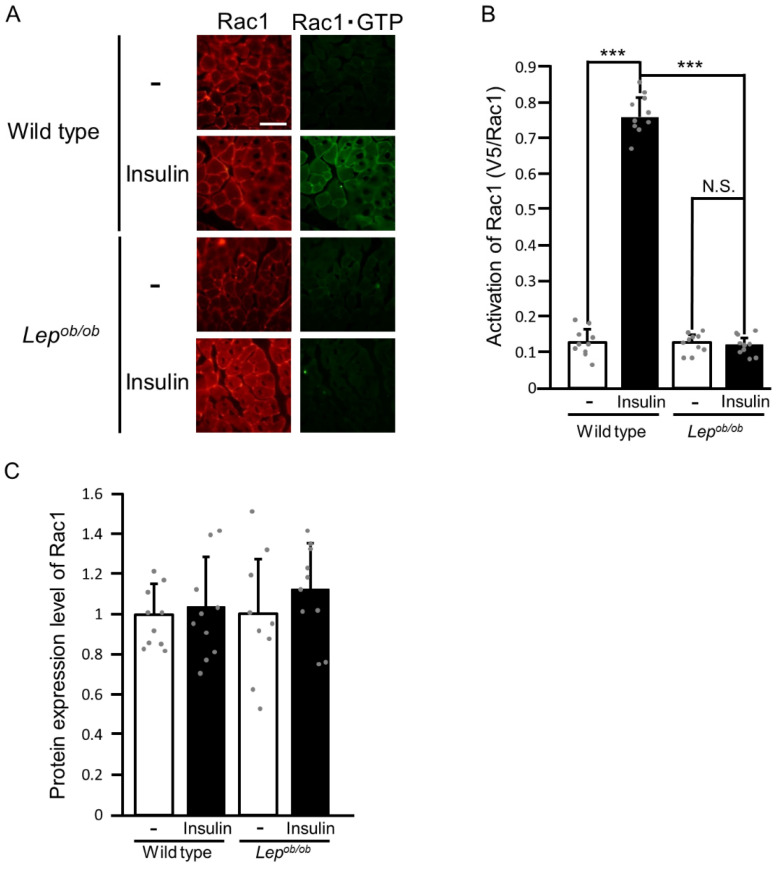
The activation of Rac1 following insulin stimulation in the gastrocnemius muscle of *Lep^ob/ob^* mice. (**A**) Insulin was administered by intravenous injection, and Rac1 activation in gastrocnemius muscle was assessed by using the activation-specific probe glutathione S-transferase (GST)-POSH(251-489)-V5×3. Total (red color) and GTP-bound activated (green color) Rac1 molecules in frozen sections of gastrocnemius muscle were visualized. Scale bar, 100 μm. (**B**) Quantification of the activation of Rac1 (V5/Rac1, arbitrary units). Data are shown as means ± S.E. (*n* = 10). *** *p* < 0.001 (Student’s *t*-test). (**C**) Quantification of the protein expression level of Rac1 relative to that in unstimulated wild-type muscle. Data are shown as means ± S.E. (*n* = 10).

We next evaluated the activation state of the endogenous Rac1 protein in insulin-stimulated and unstimulated gastrocnemius muscle (Figure 2). We first quantified the protein expression level of endogenous Rac1 in insulin-stimulated and unstimulated gastrocnemius muscle of wild-type and *Lep^ob/ob^* mice and found no statistically significant difference (Figure 2A,C). The GTP-bound activated form of Rac1 in cross-sections of gastrocnemius muscle isolated from insulin-stimulated and unstimulated mice was specifically recognized by an activation-specific polypeptide probe consisting of an epitope tag and the Rac1-binding domain of the target protein POSH [15,16,19,20]. This probe was then detected by immunofluorescent microscopy by using the anti-tag antibody. The specificity of the probe was demonstrated in Supplemental Figure S1A. Intravenous administration of insulin increased the level of the GTP-bound activated form of Rac1 in the gastrocnemius muscle of wild-type mice, whereas insulin showed virtually no effect on the activation state of Rac1 in the gastrocnemius muscle of *Lep^ob/ob^* mice (Figure 2A,B). These results are of significance, providing direct evidence for impaired Rac1 activation in response to insulin in the skeletal muscle of obese mice.

**Figure 3:**
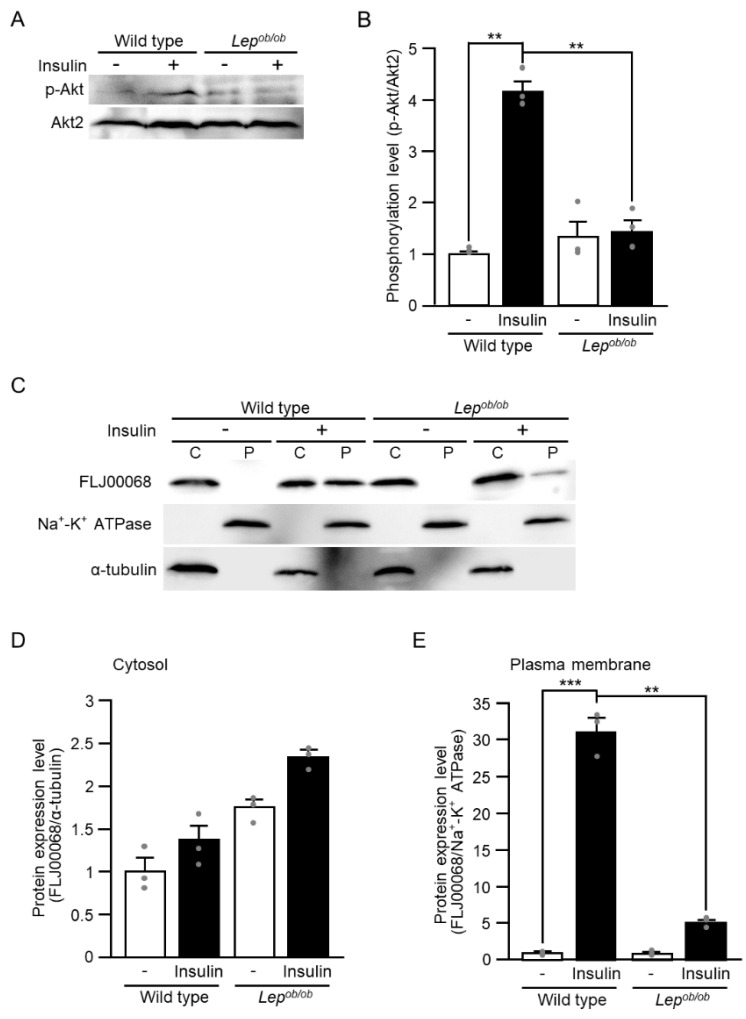
The activation of upstream regulators for Rac1 following insulin stimulation in the gastrocnemius muscle of *Lep^ob/ob^* mice. (**A**) Insulin was administered by intravenous injection, and the phosphorylation of serine in the C-terminal hydrophobic motif of Akt proteins (serine-474 of Akt2) in gastrocnemius muscle was assessed by immunoblot analysis. Total Akt2 and phosphorylated Akt molecules were detected. (**B**) Quantification of the phosphorylation level of Akt (p-Akt/Akt2) relative to that in unstimulated wild-type muscle. Data are shown as means ± S.E. (*n* = 3). ** *p* < 0.01 (Student’s *t*-test). (**C**) Insulin was administered by intravenous injection, and the translocation of FLJ00068 to the plasma membrane in gastrocnemius muscle was assessed by immunoblot analysis. FLJ00068 molecules in cytosolic (C) and plasma membrane (P) fractions were detected. (**D**) Quantification of the protein expression level of FLJ00068 in the cytosol (FLJ00068/α-tubulin) relative to that in unstimulated wild-type muscle. Data are shown as means ± S.E. (*n* = 3). (**E**) Quantification of the protein expression level of FLJ00068 in the plasma membrane (FLJ00068/Na^+^-K^+^-ATPase) relative to that in unstimulated wild-type muscle. Data are shown as means ± S.E. (*n* = 3). ** *p* < 0.01, *** *p* < 0.001, (Student’s *t*-test).

We previously investigated the mechanisms underlying insulin-induced Rac1 activation, showing that the protein kinase Akt2 and the GEF FLJ00068 served as regulators for Rac1 activation downstream of the insulin receptor [11,13,14,16,19,20]. Considering these mechanisms, we next examined the insulin-stimulated activation of Akt2 and FLJ00068. The activity of Akt proteins is known to be controlled via phosphorylation of a specific serine residue in the C-terminal hydrophobic motif (serine-474 of Akt2) and, therefore, we examined phosphorylation of this serine residue in gastrocnemius muscle following intravenous injection of insulin by immunoblotting using a phospho-specific antibody (Figure 3A,B). In wild-type mice, this serine residue was phosphorylated in response to insulin, whereas insulin did not increase the phosphorylation level in *Lep^ob/ob^* mice (Figure 3A,B). We then examined insulin-dependent translocation of the GEF FLJ00068 to the plasma membrane in the gastrocnemius muscle of wild-type and *Lep^ob/ob^* mice since plasma membrane translocation of FLJ00068 is thought to be a trigger of its activation [20]. Following the intravenous injection of insulin, only a small increase in the level of plasma membrane-localized FLJ00068 was observed in *Lep^ob/ob^* mice in contrast to wild-type mice, in which plasma membrane translocation of FLJ00068 was highly induced (Figure 3C–E). Taken together, failure of insulin to induce the activation of Rac1 in the gastrocnemius muscle of *Lep^ob/ob^* mice may be ascribed to impaired activation of Akt2 and subsequent less responsiveness of FLJ00068 to insulin stimulation.

**Figure 4:**
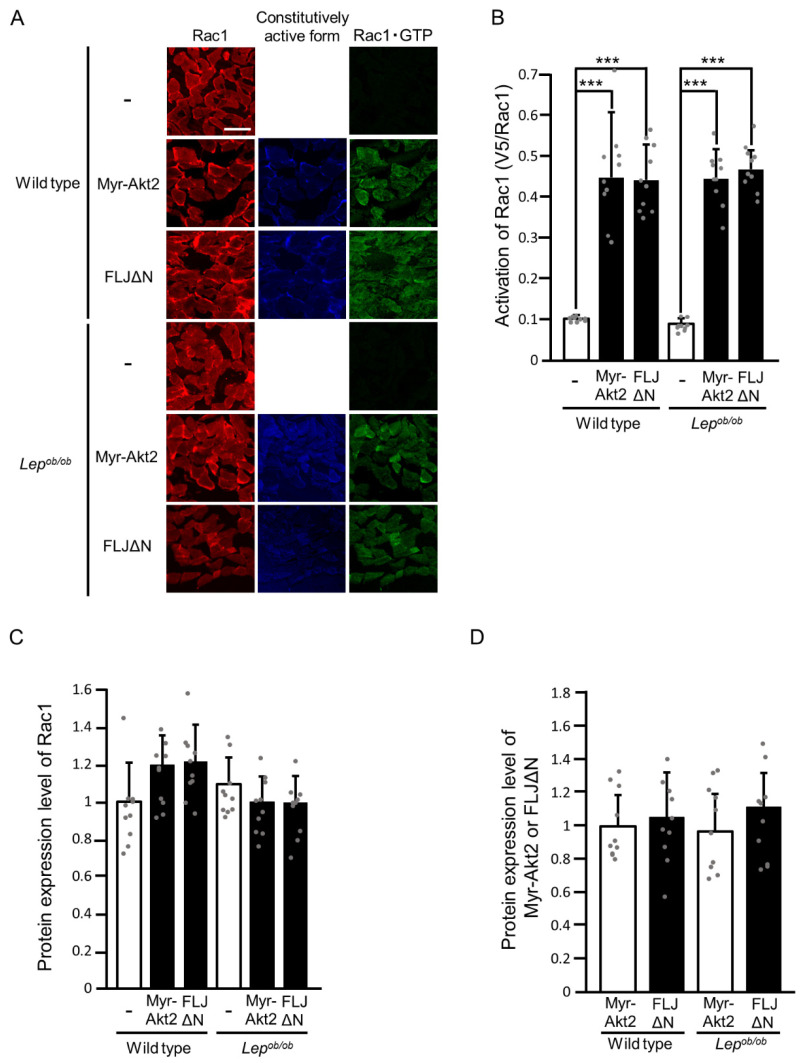
The activation of Rac1 following the ectopic expression of constitutively activated forms of Akt2 (Myr-Akt2) and FLJ00068 (FLJΔN) in the gastrocnemius muscle of *Lep^ob/ob^* mice. (**A**) Myr-Akt2 and FLJΔN (blue color) were ectopically expressed in gastrocnemius muscle, and Rac1 activation was assessed by using the activation-specific probe GST-POSH(251-489)-V5×3. Total (red color) and GTP-bound activated (green color) Rac1 molecules in frozen sections of gastrocnemius muscle were visualized. Scale bar, 100 μm. (**B**) Quantification of the activation of Rac1 (V5/Rac1, arbitrary units). Data are shown as means ± S.E. (*n* = 10). *** *p* < 0.001 (Student’s *t*-test). (**C**) Quantification of the protein expression level of Rac1 relative to that in unstimulated wild-type muscle. Data are shown as means ± S.E. (*n* = 10). (**D**) Quantification of protein expression levels of Myr-Akt2 and FLJΔN relative to the level of Myr-Akt2 in wild-type muscle. Data are shown as means ± S.E. (*n* = 10).

To further confirm that signal transduction for Rac1 activation downstream of Akt2 is intact in *Lep^ob/ob^* mouse skeletal muscle, we next tested the ability of constitutively activated forms of Akt2 and FLJ00068 (N-terminally myristoylated Akt2 (Myr-Akt2) [19] and N-terminally truncated FLJ00068 (FLJΔN) [19], respectively) to induce Rac1 activation (Figure 4). Both of the constitutively activated forms, when ectopically expressed, induced the activation of Rac1, as determined by the overlay assay using the activation-specific polypeptide probe described above, in the gastrocnemius muscle of not only wilt-type but also *Lep^ob/ob^* mice (Figure 4A,B). Hence, it is plausible that mechanisms whereby Akt2 causes the activation of Rac1 are not affected by obesity. There was no significant difference in the protein expression level of endogenous Rac1 among stimulated and unstimulated gastrocnemius muscle of wild-type and *Lep^ob/ob^* mice (Figure 4A,C). Furthermore, protein expression levels of Myr-Akt2 and FLJΔN in wild-type gastrocnemius muscle were considered equivalent to those in *Lep^ob/ob^* gastrocnemius muscle (Figure 4A,D). No substantial damage to plasma membrane structures due to electroporation procedures was detected by immunofluorescent staining with an anti-Na^+^-K^+^ ATPase antibody (Supplemental Figure S2).

### 2.2. Partial Inhibition of RalA Activation Downstream of Rac1 in Lep^ob/ob^ Mouse Skeletal Muscle

**Figure 5:**
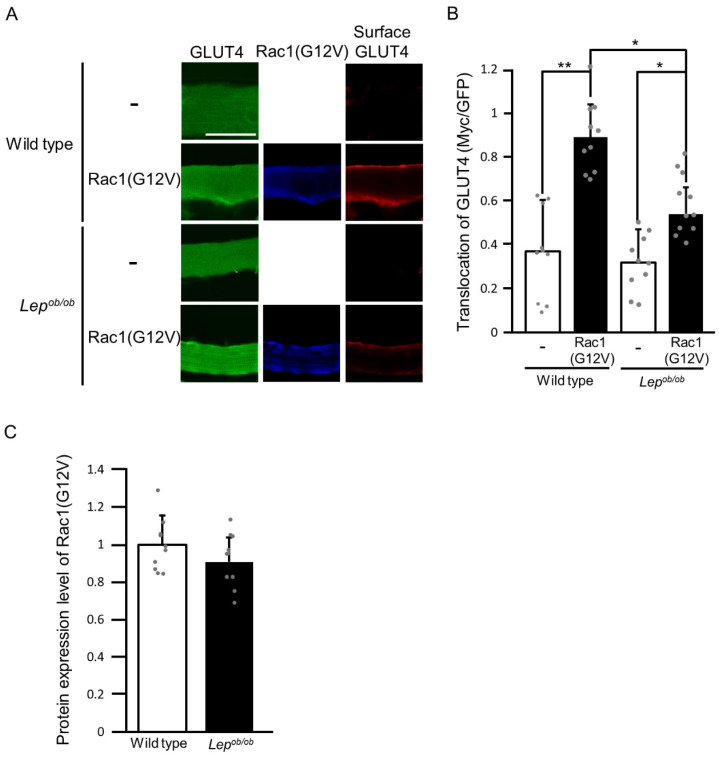
The translocation of GLUT4 to the plasma membrane following the ectopic expression of the constitutively activated form of Rac1 (Rac1(G12V)) in the gastrocnemius muscle of *Lep^ob/ob^* mice. (**A**) Rac1(G12V) (blue color) was ectopically expressed in gastrocnemius muscle, and GLUT4 translocation was assessed by using the exofacial epitope-tagged GLUT4 reporter GLUT4*myc*7-GFP. Total (green color) and cell surface-localized (red color) GLUT4*myc*7-GFP molecules in gastrocnemius muscle fibers were visualized. Scale bar, 50 μm. (**B**) Quantification of the translocation of GLUT4 to the plasma membrane (Myc/GFP, arbitrary units). Data are shown as means ± S.E. (*n* = 10). * *p* < 0.05, ** *p* < 0.01 (Student’s *t*-test). (**C**) Quantification of the protein expression level of Rac1(G12V) relative to that in wild-type muscle. Data are shown as means ± S.E. (*n* = 10).

The effect of obesity on signal transduction downstream of Rac1 for the induction of GLUT4 translocation was then investigated. As a first step, GLUT4 translocation induced by a constitutively activated mutant of Rac1, Rac1(G12V), was examined (Figure 5). Interestingly, Rac1(G12V)-induced GLUT4 translocation was partially abrogated in the gastrocnemius muscle of *Lep^ob/ob^* mice compared to wild-type mice (Figure 5A,B). This finding implies that signal transduction downstream of Rac1 is also impaired in *Lep^ob/ob^* mouse skeletal muscle. The protein expression level of Rac1(G12V) in wild-type gastrocnemius muscle was considered equivalent to that in *Lep^ob/ob^* gastrocnemius muscle (Figure 5A,C).

**Figure 6:**
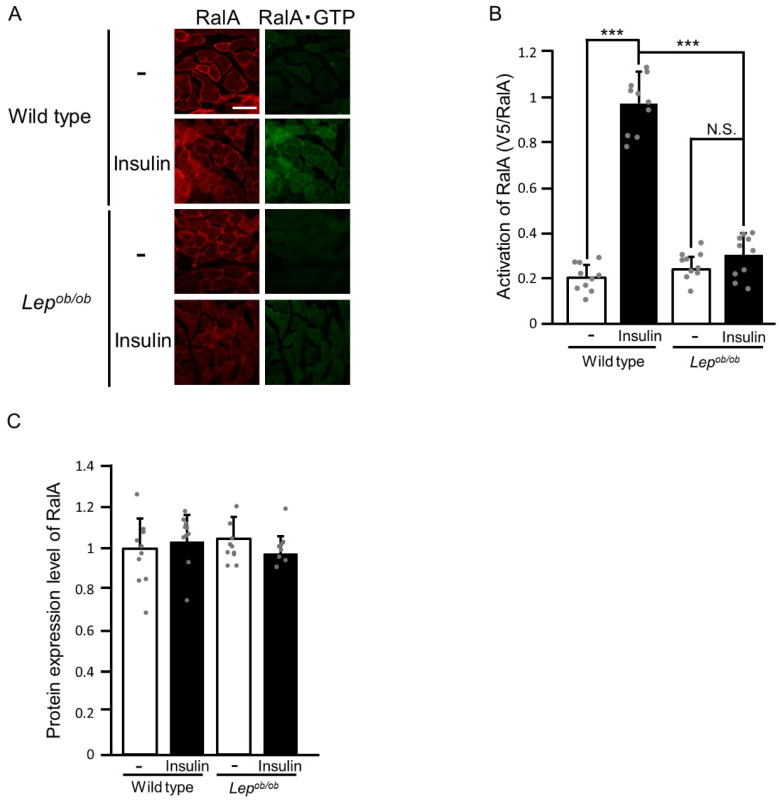
The activation of RalA following insulin stimulation in the gastrocnemius muscle of *Lep^ob/ob^* mice. (**A**) Insulin was administered by intravenous injection, and RalA activation in gastrocnemius muscle was assessed by using the activation-specific probe GST-V5×3-Sec5(1-99). Total (red color) and GTP-bound activated (green color) RalA molecules in frozen sections of gastrocnemius muscle were visualized. Scale bar, 100 μm. (**B**) Quantification of the activation of RalA (V5/RalA, arbitrary units). Data are shown as means ± S.E. (*n* = 10). *** *p* < 0.001 (Student’s *t*-test). (**C**) Quantification of the protein expression level of RalA relative to that in unstimulated wild-type muscle. Data are shown as means ± S.E. (*n* = 10).

To further address this issue, we next examined the activation state of the Ras family GTPase RalA, which is also known to serve as a critical regulator of GLUT4 translocation downstream of Rac1 in skeletal muscle [16,22,23]. The activation state of RalA was assessed by the overlay assay employing a polypeptide probe that specifically binds to the activated form of RalA (RalA·GTP), similar to that for Rac1 [16,22,23]. The specificity of the probe was demonstrated in Supplemental Figure S1B. First, we examined the activation of RalA in the gastrocnemius muscle of wild-type and *Lep^ob/ob^* mice following insulin injection (Figure 6). Insulin-stimulated RalA activation was almost completely inhibited in *Lep^ob/ob^* mice, as anticipated from the above-described results that insulin failed to yield the activated form of Rac1, which is required for the activation of RalA (Figure 6A,B). As in the case of Rac1, there was no significant difference in the protein expression level of endogenous RalA among insulin-stimulated and unstimulated gastrocnemius muscle of wild-type and *Lep^ob/ob^* mice (Figure 6A,C).

**Figure 7:**
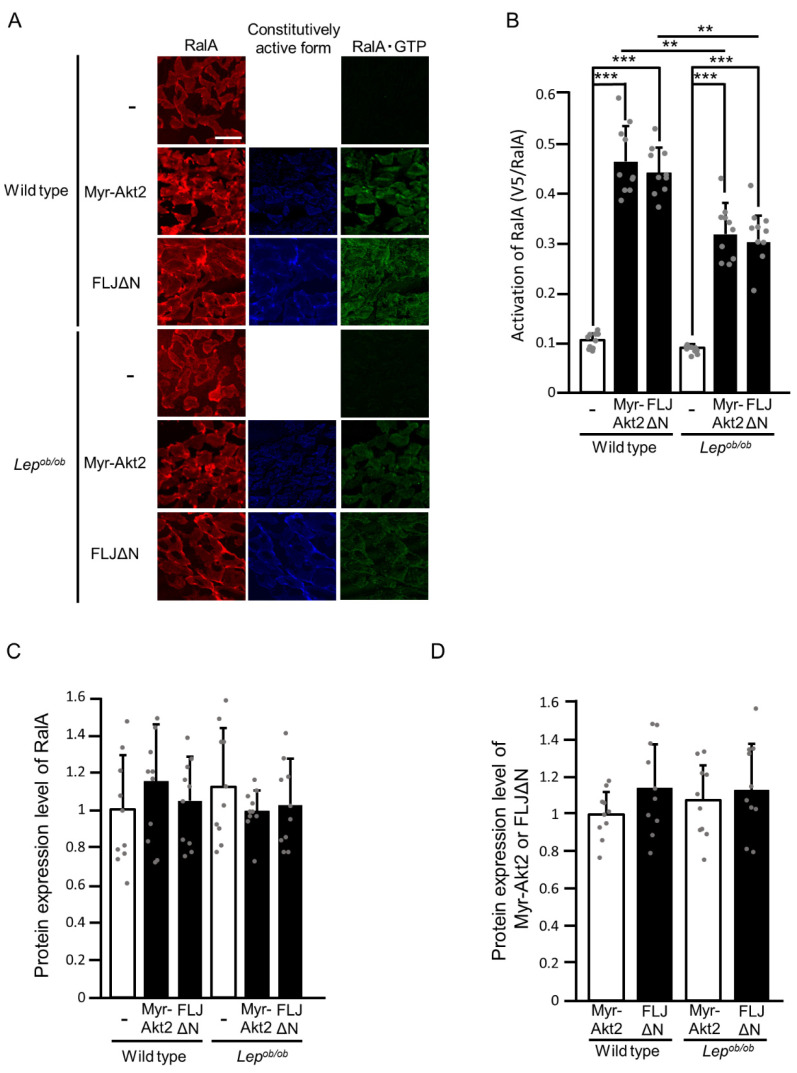
The activation of RalA following the ectopic expression of constitutively activated forms of Akt2 (Myr-Akt2) and FLJ00068 (FLJΔN) in the gastrocnemius muscle of *Lep^ob/ob^* mice. (**A**) Myr-Akt2 and FLJΔN (blue color) were ectopically expressed in gastrocnemius muscle, and RalA activation was assessed by using the activation-specific probe GST-V5×3-Sec5(1-99). Total (red color) and GTP-bound activated (green color) RalA molecules in frozen sections of gastrocnemius muscle were visualized. Scale bar, 100 μm. (**B**) Quantification of the activation of RalA (V5/RalA, arbitrary units). Data are shown as means ± S.E. (*n* = 10). ** *p* < 0.01, *** *p* < 0.001 (Student’s *t*-test). (**C**) Quantification of the protein expression level of RalA relative to that in unstimulated wild-type muscle. Data are shown as means ± S.E. (*n* = 10). (**D**) Quantification of protein expression levels of Myr-Akt2 and FLJΔN relative to the level of Myr-Akt2 in wild-type muscle. Data are shown as means ± S.E. (*n* = 10).

Subsequently, we examined whether constitutively activated forms of Akt2 and FLJ00068 could induce RalA activation (Figure 7). Both of the constitutively activated mutants in fact induced RalA activation in the gastrocnemius muscle of wild-type mice, whereas RalA activations by these mutants were significantly compromised in *Lep^ob/ob^* mice (Figure 7A,B). There was no significant difference in the protein expression level of endogenous RalA among stimulated and unstimulated gastrocnemius muscle of wild-type and *Lep^ob/ob^* mice (Figure 7A,C). Furthermore, protein expression levels of Myr-Akt2 and FLJΔN in wild-type gastrocnemius muscle were considered equivalent to those in *Lep^ob/ob^* gastrocnemius muscle (Figure 7A,D).

**Figure 8:**
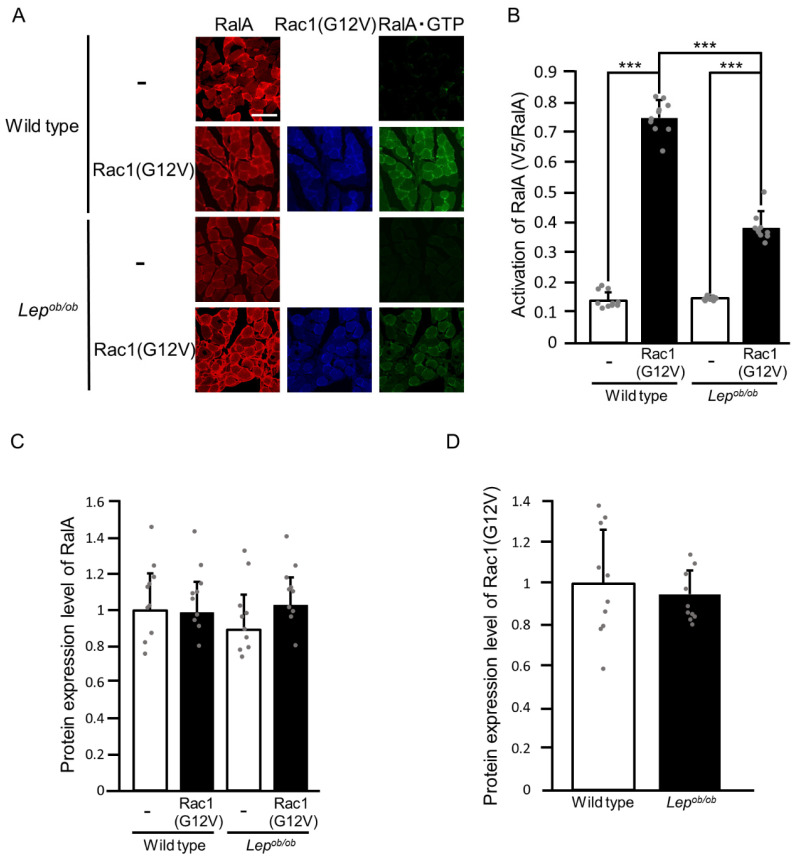
The activation of RalA following the ectopic expression of the constitutively activated form of Rac1 (Rac1(G12V)) in the gastrocnemius muscle of *Lep^ob/ob^* mice. (**A**) Rac1(G12V) (blue color) was ectopically expressed in gastrocnemius muscle, and RalA activation was assessed by using the activation-specific probe GST-V5×3-Sec5(1-99). Total (red color) and GTP-bound activated (green color) RalA molecules in frozen sections of gastrocnemius muscle were visualized. Scale bar, 100 μm. (**B**) Quantification of the activation of RalA (V5/RalA, arbitrary units). Data are shown as means ± S.E. (*n* = 10). *** *p* < 0.001 (Student’s *t*-test). (**C**) Quantification of the protein expression level of RalA relative to that in unstimulated wild-type muscle. Data are shown as means ± S.E. (*n* = 10). (**D**) Quantification of the protein expression level of Rac1(G12V) relative to that in wild-type muscle. Data are shown as means ± S.E. (*n* = 10).

Furthermore, we evaluated RalA activation upon ectopic expression of the constitutively activated form of Rac1, Rac1(G12V) (Figure 8). The ectopic expression of Rac1(G12V) actually caused RalA activation in the gastrocnemius muscle of wild-type mice (Figure 8A,B). RalA was also activated by ectopically expressed Rac1(G12V) in the gastrocnemius muscle of *Lep^ob/ob^* mice, but the activation level was significantly lower than in the case of wild-type mice (Figure 8A,B). There was no significant difference in the protein expression level of endogenous RalA among stimulated and unstimulated gastrocnemius muscle of wild-type and *Lep^ob/ob^* mice (Figure 8A,C). The protein expression level of Rac1(G12V) in wild-type gastrocnemius muscle was considered equivalent to that in *Lep^ob/ob^* gastrocnemius muscle (Figure 8A,D).

**Figure 9:**
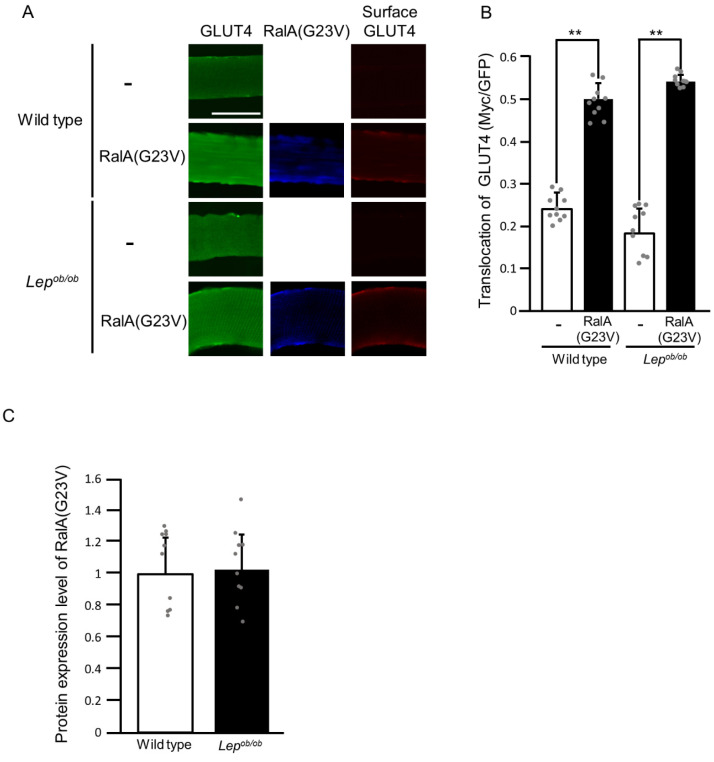
The translocation of GLUT4 to the plasma membrane following the ectopic expression of the constitutively activated form of RalA (RalA(G23V)) in the gastrocnemius muscle of *Lep^ob/ob^* mice. (**A**) RalA(G23V) (blue color) was ectopically expressed in gastrocnemius muscle, and GLUT4 translocation was assessed by using the exofacial epitope-tagged GLUT4 reporter GLUT4*myc*7-GFP. Total (green color) and cell surface-localized (red color) GLUT4*myc*7-GFP molecules in gastrocnemius muscle fibers were visualized. Scale bar, 50 μm. (**B**) Quantification of the translocation of GLUT4 to the plasma membrane (Myc/GFP, arbitrary units). Data are shown as means ± S.E. (*n* = 10). ** *p* < 0.01 (Student’s *t*-test). (**C**) Quantification of the protein expression level of RalA(G23V) relative to that in wild-type muscle. Data are shown as means ± S.E. (*n* = 10).

Lastly, we examined whether signal transduction for GLUT4 translocation downstream of RalA is influenced by obesity. The constitutively activated mutant of RalA, RalA(G23V), was ectopically expressed in the gastrocnemius muscle of wild-type and *Lep^ob/ob^* mice, and then plasma membrane translocation of GLUT4 was assessed by using the exofacial epitope-tagged GLUT4 reporter as described above (Figure 9). RalA(G23V), when ectopically expressed, induced GLUT4 translocation to the plasma membrane in the gastrocnemius muscle of wild-type mice, and this effect was not impaired in *Lep^ob/ob^* mice (Figure 9A,B). The protein expression level of RalA(G23V) in wild-type gastrocnemius muscle was considered equivalent to that in *Lep^ob/ob^* gastrocnemius muscle (Figure 9A,C). Collectively, signal transduction for the regulation of RalA downstream of Rac1 was partially inhibited in *Lep^ob/ob^* mice, whereas the regulation of GLUT4 translocation downstream of RalA was not affected by obesity.

## 3. Discussion

In this study, we investigated whether Rac1-mediated signaling that regulates glucose uptake in skeletal muscle was affected by obesity by employing the *Lep^ob/ob^* mouse model. We demonstrated that insulin-induced Rac1 activation was severely impaired in the gastrocnemius muscle of *Lep^ob/ob^* mice, presumably due to the impairment of Akt2 phosphorylation and the subsequent plasma membrane translocation of the Rac1 regulator FLJ00068. Considering that Rac1 is an essential component of insulin signaling that regulates glucose uptake, it is likely that the failure of Rac1 activation is a major cause of the unresponsiveness of GLUT4 to insulin stimulation in *Lep^ob/ob^* mice. On the other hand, it is also possible that the activation of signaling molecules other than Rac1, such as AS160 and Rab proteins [4,5,6], is also impaired downstream of Akt2, leading to the attenuation of GLUT4 translocation in *Lep^ob/ob^* gastrocnemius muscle. Additionally, we showed that signal transduction for the regulation of RalA downstream of Rac1 was partially impaired in *Lep^ob/ob^* mice, whereas signaling downstream of RalA that led to GLUT4 translocation was unaffected by obesity. The finding that signaling downstream of Rac1 remains functional, at least in part, even in *Lep^ob/ob^* gastrocnemius muscle is important, implying that glucose uptake can be restored by the activation of Rac1 in response to upstream signals other than insulin (see below). Studies using other mouse models of obesity are also considered important to further confirm the above conclusions in the present study. For instance, the investigation of Rac1 signaling in mice fed a high-fat diet may be crucial and will be performed in our laboratory in the future.

In a previous study, Sylow et al. reported that the increase in the phosphorylation level of the protein kinase PAK following insulin stimulation in mouse soleus and extensor digitorum longus (EDL) muscles was not observed when fed a high-fat diet [10]. Given that Rac1 is an upstream regulator of PAK, it is possible that the impaired response of PAK to insulin is ascribed to impaired Rac1 signaling in obese mice, as reported in our present study. However, the level of PAK phosphorylation after insulin stimulation in mice fed a high-fat diet was almost equivalent to that in mice fed a normal diet, and the unresponsiveness of PAK to insulin on a high-fat diet was due to an increased basal level [10]. Therefore, the profile of insulin-stimulated PAK phosphorylation in mice fed a high-fat diet is not necessarily the same as that of insulin-stimulated Rac1 activation in *Lep^ob/ob^* mice, as described in this study.

A slight decrease in the expression level of Rac1 was reported in the soleus, but not the gastrocnemius, muscle of *Lep^ob/ob^* mice [18]. Rac1 expression was also reduced in EDL muscle on a high-fat diet [10]. On the other hand, we could not detect any significant reduction in the level of Rac1 expression in the gastrocnemius muscle of *Lep^ob/ob^* mice (Figure 2 and Figure 4) in accordance with the above study [18]. Therefore, obesity may cause a decrease in the Rac1 protein level only in specific types of skeletal muscles, such as soleus and EDL muscles, which may partly account for insulin resistance in obese mice.

In addition to insulin stimulation, the decrease of the intracellular ATP level following muscle contraction enhances glucose uptake in skeletal muscle [26,27]. The decrease of the intracellular ATP level is sensed by a protein kinase called AMP-activated protein kinase (AMPK), which in turn stimulates the downstream signaling pathway that regulates GLUT4 translocation to the plasma membrane [26,27]. Although the detailed mechanisms for the induction of GLUT4 translocation by activated AMPK remain incompletely understood, Akt2 does not seem to be involved in this process [26,27]. Interestingly, Rac1 is also responsible, at least in part, for AMPK-dependent GLUT4 translocation in skeletal muscle [28,29,30,31,32,33]. It is plausible that the activity of Rac1 is regulated downstream of AMPK by mechanisms other than those in insulin signaling involving Akt2, and therefore, Rac1 activation downstream of AMPK may not be impaired by obesity. Moreover, the involvement of Rac1 in AMPK-independent glucose uptake signaling induced by contraction was reported in mouse EDL muscle [34]. Contraction-stimulated, AMPK-independent mechanisms for Rac1 activation may also be different from those in insulin signaling and may not be inhibited by obesity.

In this study, we provided evidence that signaling for GLUT4 translocation downstream of Rac1 was only partly impaired in the skeletal muscle of *Lep^ob/ob^* mice (Figure 5, Figure 7 and Figure 8). Namely, glucose uptake actually occurred once Rac1 was sufficiently activated even in the skeletal muscle of *Lep^ob/ob^* mice. Given these findings, it is plausible that contraction-induced glucose uptake is not fully inhibited in the skeletal muscle of obese individuals. This is important because it supports the notion that exercise is effective in improving glucose uptake in skeletal muscle, even in insulin-resistant states.

A variety of cytokines, such as tumor necrosis factor α, are known to negatively regulate insulin signaling in skeletal muscle [35]. These cytokines may also affect Rac1-mediated signaling via the inhibition of Akt2. It is also possible that fatty acids produced in response to hyperphagia may cause the unresponsiveness of Rac1 to insulin stimulation. To examine these possibilities, we will further analyze Rac1 signaling not only in *Lep^ob/ob^* mice but also in mice fed a high-fat diet. Here, we reported that signal transduction downstream of Rac1 was also inhibited in *Lep^ob/ob^* mouse gastrocnemius muscle. It remains unclear whether common extracellular factors are responsible for the negative effects on signal transduction upstream and downstream of Rac1. In addition, the mechanisms whereby Rac1 activates RalA are largely unknown, and therefore the protein that the inhibitory signal targets in *Lep^ob/ob^* mice remains obscure. Further studies are needed to determine the target of obesity-induced negative regulation downstream of Rac1.

## 4. Materials and Methods

### 4.1. Materials

A rat monoclonal antibody against the hemagglutinin (HA) epitope tag (11 867 423 001), a mouse monoclonal antibody against the Myc epitope tag (06-549), and goat polyclonal antibody against the V5 epitope tag (A190-119A) were purchased from Roche Applied Science (Penzberg, Germany), Merck Millipore (Burlington, MA, USA), and Bethyl Laboratories (Montgomery, TX, USA), respectively. Mouse monoclonal antibodies against Rac1 (610651) and RalA (610221) were purchased from BD Biosciences (Franklin Lakes, NJ, USA). Antibodies against goat IgG, mouse IgG, rabbit IgG, and rat IgG conjugated with CF™ 350/543/647 were purchased from Biotium (Fremont, CA, USA). Insulin was purchased from Eli Lilly (Indianapolis, IN, USA). A rabbit polyclonal antibody against FLJ00068 (ab137898) was purchased from Abcam (Cambridge, UK). Mouse monoclonal antibodies against α-tubulin (T9026) and Na^+^-K^+^ ATPase (05-369) were purchased from SIGMA-Aldrich (St. Louis, MO, USA). A goat polyclonal antibody against Akt2 (AF23151) was purchased from R&D systems (Minneapolis, MN, USA). A mouse monoclonal antibody against phospho-(Ser473) Akt (4051) was purchased from Cell Signaling Technology (Danvers, MA, USA). A sheep polyclonal antibody against mouse IgG (NA931) conjugated with horseradish peroxidase (HRP) was purchased from Cytiva (Emeryville, MA, USA). A donkey polyclonal antibody against rabbit IgG (w4018) conjugated with HRP was purchased from Promega (Madison, WI, USA). A donkey polyclonal antibody against goat IgG (sc-2020) conjugated with HRP was purchased from Santa Cruz Biotechnology (Santa Cruz, CA, USA).

### 4.2. Animal Experiments

All animal experiments were approved by the Ethics Committee for Animal Experiments at Osaka Metropolitan University (Approval Code: #20-84, #21-88, and #22-106) and carried out according to the institutional guidelines of Osaka Metropolitan University. All mice used in this study are on the C57BL/6 genetic background. *Lep^ob/ob^* mice were purchased from Japan SLC (Hamamatsu, Japan). Mice were fed a normal chow diet, and adult (22 to 26-week-old) male mice were used for all experiments.

### 4.3. Gene Transfer into Mouse Gastrocnemius Muscle by Electroporation

Plasmid DNAs were introduced into mouse gastrocnemius muscle by electroporation as previously described [16,20]. Mice were anesthetized by intraperitoneal injection of a solution of medetomidine (0.3 mg/kg of body weight), midazolam (4.0 mg/kg of body weight), and butorphanol (5.0 mg/kg of body weight). A combination of expression plasmids (pCAGGS-GLUT4*myc*7-GFP, pCAGGS-Myr-Akt2-HA×3, pCAGGS-HA×2-FLJ68ΔN, pCAGGS-HA×3-Rac1(G12V), and pCAGGS-HA×3-RalA(G23V)) (80 µg in total) were dissolved in 50 µL of 9 mg/mL NaCl and injected longitudinally into gastrocnemius muscle with a 27-gauge needle. A pair of stainless steel electrode needles fixed 5 mm apart were then inserted into the muscle belly, and square wave electrical pulses (50 milliseconds) were applied three times (100 V, 90 V, and 81 V, respectively) at 100-millisecond intervals (for poring) using a pulse generator (NEPA21 Type II, Nepa Gene (Ichikawa, Japan)). Subsequently, square wave electrical pulses (50 milliseconds) were applied three times (20 V, 12 V, and 7.2 V, respectively) at 100-millisecond intervals, followed by three more pulses under the same conditions except that the polarity is opposite (for transfer).

### 4.4. Isolation of Mouse Gastrocnemius Muscle Fibers and the GLUT4 Reporter Assay

Mouse gastrocnemius muscle fibers were isolated, and a GLUT4 reporter assay was performed as previously described [12,14,16,19,20,23]. The GLUT4 reporter GLUT4*myc*7-GFP was originally described in [25]. Mice were fasted for 16 h and anesthetized by intraperitoneal injection of a solution of medetomidine (0.3 mg/kg of body weight), midazolam (4.0 mg/kg of body weight), and butorphanol (5.0 mg/kg of body weight) 5 days after gene transfer by electroporation. Insulin (175.5 µg/kg of body weight) was then administered intravenously. Gastrocnemius muscle was excised from anesthetized mice and fixed with 40 mg/mL paraformaldehyde in phosphate-buffered saline (PBS) for 40 min. Individual muscle fibers were teased from fixed muscle with fine forceps under stereomicroscopy and incubated in 2% (*v*/*v*) normal goat serum in PBS for more than 1 h. Thereafter, muscle fibers were treated with an anti-Myc tag antibody (for the detection of GLUT4*myc*7-GFP translocated to the sarcolemma) for 1 h and washed three times with PBS. Subsequently, muscle fibers were permeabilized with 0.5% (*v*/*v*) Triton X-100 in PBS for 15 min and incubated in 0.5% (*v*/*v*) Triton X-100 in PBS supplemented with 2% (*v*/*v*) normal goat serum for 1 h. Muscle fibers were then treated with antibodies against the HA tag (for the detection of Rac1(G12V) and RalA(G23V)) overnight at 4°C and washed three times with 0.1% (*v*/*v*) Tween 20 in PBS. Primary antibodies were subsequently detected with fluoresceinated secondary antibodies. Images were obtained and analyzed using a confocal laser-scanning microscope (FV1200, Olympus (Tokyo, Japan)). Fluorescent intensities were quantified using the image processing software ImageJ v1.52. The relative amount of GLUT4*myc*7-GFP translocated to the sarcolemma was estimated by the ratio of Myc and GFP fluorescent intensities (Myc/GFP). Values of 10 muscle fibers in total from three different mice under each condition were used for statistical analysis (Student’s *t*-test).

### 4.5. Detection of Activated Forms of Rac1 and RalA in Frozen Sections of Mouse Gastrocnemius Muscle

Activated forms of Rac1 and RalA were detected in frozen sections of mouse gastrocnemius muscle as previously described [16,20]. Mice were fasted for 16 h and anesthetized by intraperitoneal injection of a solution of medetomidine (0.3 mg/kg of body weight), midazolam (4.0 mg/kg of body weight), and butorphanol (5.0 mg/kg of body weight) 5 days after gene transfer by electroporation. Insulin (175.5 µg/kg of body weight) was then administered intravenously. Gastrocnemius muscle was excised from anesthetized mice, fixed in 40 mg/mL paraformaldehyde in PBS for 30 min on ice, and frozen in OCT compound (Sakura Finetek (Torrance, CA, USA)). Frozen sections were fixed in overlay assay buffer (50 mM Hepes-NaOH (pH 7.3), 150 mM NaCl, 20 mM MgCl_2_, and 0.05% (*v*/*v*) Tween 20) supplemented with 20 mg/mL paraformaldehyde on ice for 1 min and treated with GST-POSH(251–489)-V5×3 or GST-V5×3-Sec5(1–99) (10 μg/mL) in overlay assay buffer supplemented with 1% (*v*/*v*) Triton X-100 and 50 μg/mL bovine serum albumin on ice for 40 min. After washing three times with overlay assay buffer, frozen sections were fixed again in overlay assay buffer supplemented with 20 mg/mL paraformaldehyde on ice for 5 min. Fixed frozen sections were washed with PBS supplemented with 0.1% (*v*/*v*) Tween 20 three times and incubated with an antibody against the V5 tag (for the detection of GST-POSH(251–489)-V5×3 or GST-V5×3-Sec5(1–99)). Frozen sections were counterstained with antibodies against Rac1 or RalA and the HA tag (for the detection of Myr-Akt2, FLJ68ΔN, and Rac1(G12V)). Primary antibodies were subsequently detected with fluoresceinated secondary antibodies. Images were obtained and analyzed using a confocal laser-scanning microscope (FV1200, Olympus). Fluorescent intensities were quantified using the image processing software ImageJ v1.52. The activity of Rac1 or RalA was estimated by the ratio of V5 and Rac1 or RalA fluorescent intensities (V5/Rac1 or V5/RalA). Values of six frozen sections in total from three different mice under each condition were used for statistical analysis (Student’s *t*-test).

### 4.6. Immunoblot Analysis

Proteins separated by SDS-polyacrylamide gel electrophoresis were transferred onto a 0.45-μm pore-size polyvinylidene difluoride membrane (Cytiva). Membranes were incubated with primary and HRP-conjugated secondary antibodies. Specific proteins were visualized by Chemi-Lumi One Ultra (Nacalai (Kyoto, Japan)). Images were captured, and densitometric analysis was carried out by using a chemiluminescence imaging system (Ez-Capture MG, Atto (Tokyo, Japan)).

### 4.7. Subcellular Fractionation of Mouse Gastrocnemius Muscle

Mice were fasted for 16 h, and insulin (175.5 µg/kg of body weight) was administered via intravenous injection. Forty-five minutes later, the mice were euthanized, and gastrocnemius muscle was excised from these mice. Isolated gastrocnemius muscle was minced in a cold homogenization buffer (20 mM Tris-HCl (pH 7.4), 250 mM sucrose, 1 mM ethylenediaminetetraacetic acid, 1 mM ethylene glycol bis (2-amino ethylether) tetraacetic acid, 1 mM phenylmethylsulfonyl fluoride, 25 mM NaF, 1 mM Na_3_VO_4_, and protease inhibitor cocktail (Nacalai), and homogenized on ice using a Teflon pestle homogenizer (100 strokes) (AS one (Osaka, Japan)). The homogenate was centrifuged at 760× *g* for 5 min at 4 °C to remove nuclei and unbroken cells. The supernatant was further centrifuged at 136,000× *g* for 1 h at 4 °C, yielding a pellet of the crude plasma membrane fraction and a supernatant of the cytosol fraction. The pellet was resuspended in cold homogenization buffer.

## Data Availability

The data presented in this study are available on request.

## Supplementary Materials

The following supporting information can be downloaded at https://www.mdpi.com/article/10.3390/ijms241411531/s1.

## Abbreviations

AMPK	AMP-activated protein kinase
EDL	extensor digitorum longus
FLJΔN	N-terminally truncated FLJ00068
GEF	guanine nucleotide exchange factor
GFP	green fluorescent protein
GST	glutathione S-transferase
HA	hemagglutinin
HRP	horseradish peroxidase
*Lep^ob/ob^*	leptin-deficient obese
Myr-Akt2	N-terminally myristoylated Akt2
N.S.	not significant
PBS	phosphate-buffered saline
PI3K	phosphoinositide 3-kinase
