# Supplementary material for: Impaired Insulin Signaling Mediated by the Small GTPase Rac1 in Skeletal Muscle of the Leptin-Deficient Obese Mouse
Source: Int J Mol Sci. 2023 Jul 16;24(14):11531. doi: 10.3390/ijms241411531 (PMC10380855; doi:10.3390/ijms241411531)
Supplement: Supplementary file 1 [file ijms-24-11531-s001.zip › ijms-2454273-supplementary.pdf]

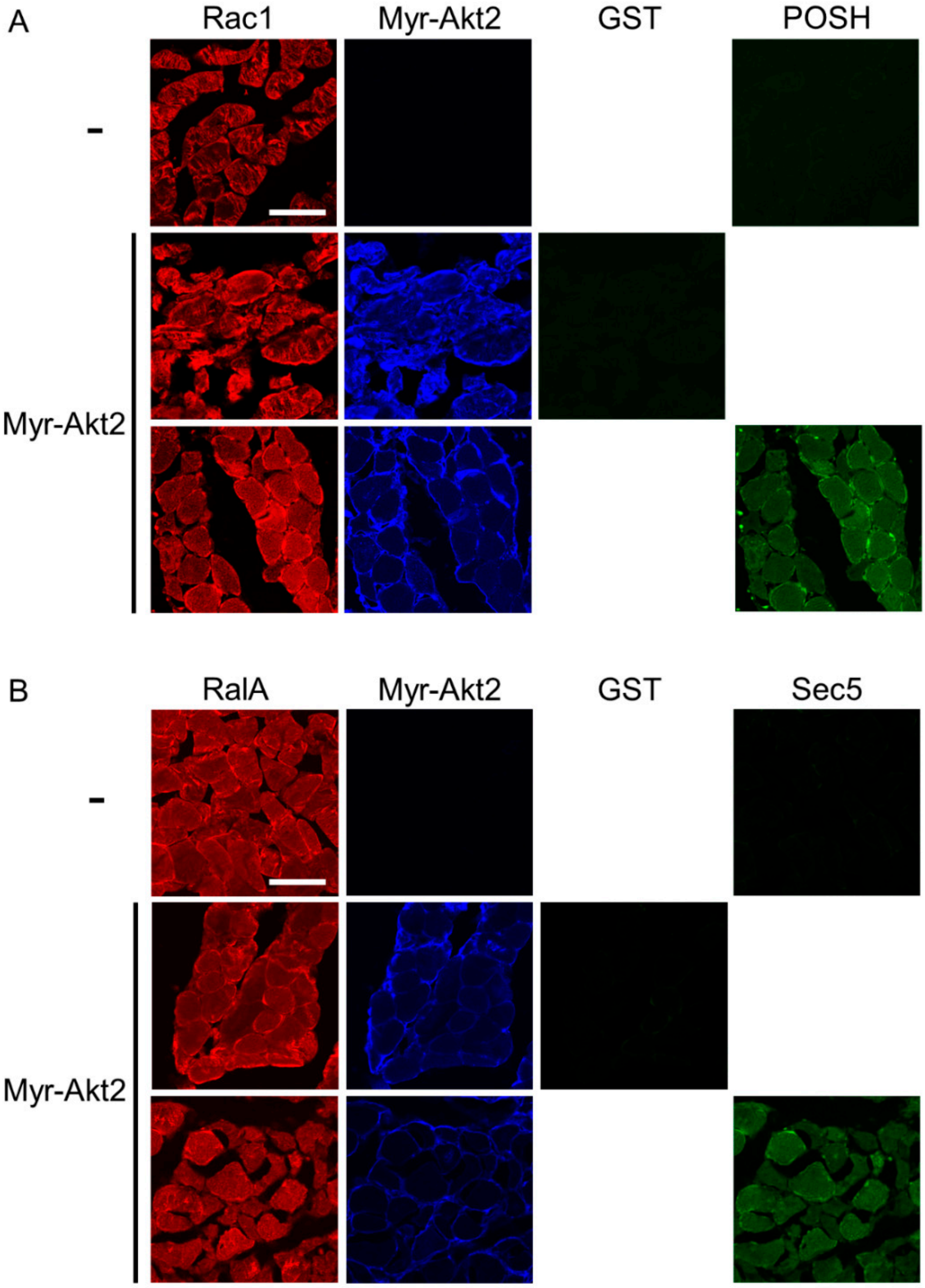

Figure S1. The detection of activated forms of Rac1 and RalA in frozen sections of gastrocnemius muscle of wild type mice following the ectopic expression of the constitutively activated form of Akt2 (Myr-Akt2). (**A**) Myr-Akt2 (blue color) was ectopically expressed in gastrocnemius muscle, and the GTP-bound activated form of Rac1 (green color) was visualized by the activation-specific probe GST-POSH(251-489)-V5×3 (POSH), but not by the control probe GST-V5×3 (GST). Total Rac1 molecules (red color) were also visualized. Scale bar, 100 μm. (**B**) Myr-Akt2 (blue color) was ectopically expressed in gastrocnemius muscle, and the GTP-bound activated form of RalA (green color) was visualized by the activation-specific probe GST-V5×3-Sec5(1-99) (Sec5), but not by the control probe GST-V5×3 (GST). Total RalA molecules (red color) were also visualized. Scale bar, 100 μm.

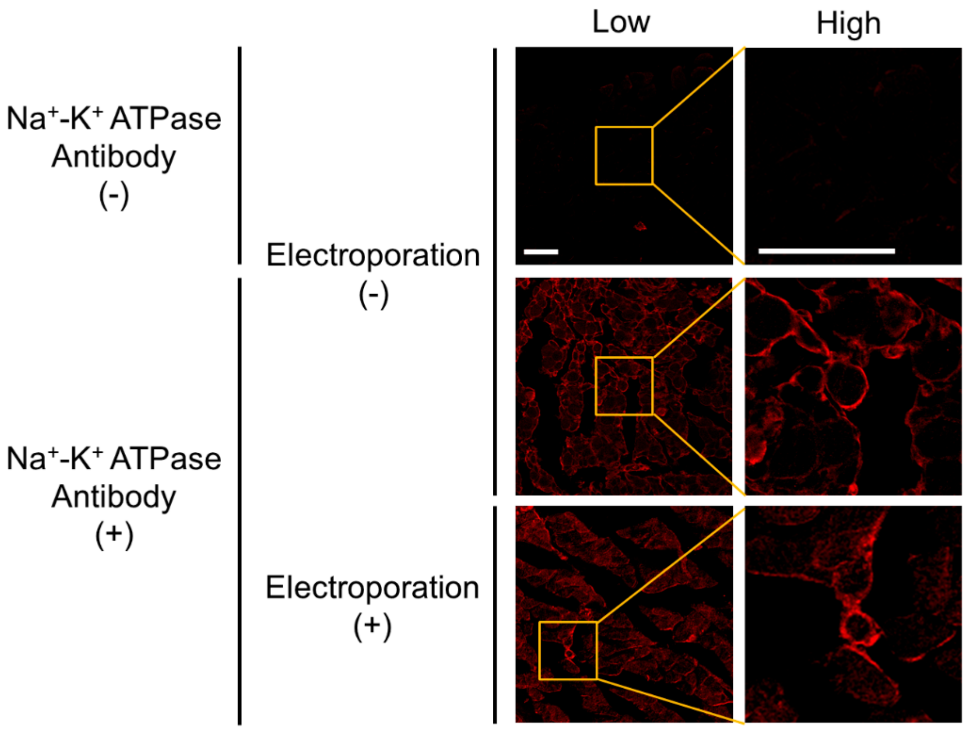

Figure S2. Plasma membrane structures of electroporated and non-electroporated gastrocnemius muscle. Plasma membranes were visualized in frozen sections of electroporated (+) and non-electroporated (−) gastrocnemius muscle with an anti-Na^{+}-K^{+} ATPase antibody. Low- and high-magnification images are shown. Scale bar, 100 μm.
